# Short-term auditory priming in freely-moving mice

**DOI:** 10.1016/j.isci.2023.107847

**Published:** 2023-09-07

**Authors:** Shir Sivroni, Hadas E. Sloin, Eran Stark

**Affiliations:** 1 Department of Physiology and Pharmacology, Faculty of Medicine, Tel Aviv University, Tel-Aviv 6997801, Israel; 2 Department of Mathematics, Afeka-Tel Aviv College of Engineering, Tel-Aviv 6910717, Israel; 3 Department of Mathematics, The Open University of Israel, Ra’anana 4353701, Israel; 4 Sagol School of Neuroscience, Tel Aviv University, Tel-Aviv 6997801, Israel; 5 Sagol Department of Neurobiology, Haifa University, Haifa 3103301, Israel

**Keywords:** Behavioral neuroscience, Sensory neuroscience

## Abstract

Priming, a change in the mental processing of a stimulus as a result of prior encounter with a related stimulus, has been observed repeatedly and studied extensively in humans. Yet currently, there is no behavioral model of short-term priming in lab animals, precluding research on the neurobiological basis of priming. Here, we describe an auditory discrimination paradigm for studying response priming in freely moving mice. We find a priming effect in success rate in all mice tested on the task. In contrast, we do not find a priming effect in response times. Compared to non-primed discrimination trials, the addition of incongruent prime stimuli reduces success rate more than congruent prime stimuli, suggesting a cognitive mechanism based on differential interference. The results establish the short-term priming phenomenon in rodents, and the paradigm opens the door to studying the cellular-network basis of priming.

## Highlights

• An auditory discrimination task is used to study short-term priming in mice
• Mice show a priming effect in success rates but not in response times
• Incongruent prime stimuli interfere with performance
• The priming phenomenon is not limited to humans

## Graphical abstract

**Figure:**
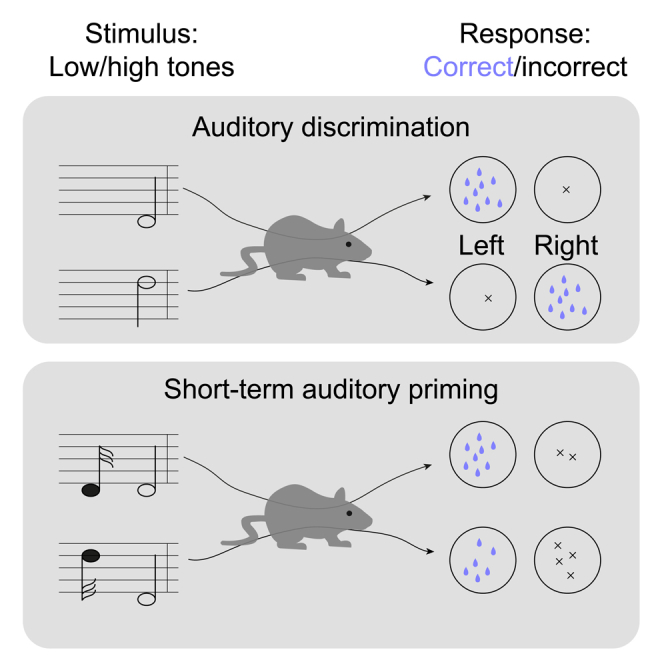

## Introduction

We are all familiar with the feeling that related but irrelevant experiences unintentionally influence our decisions. The way products, ideas, and behaviors are promoted or presented affects our tendencies or desires.^1^^,^^2^^,^^3^ For example, if you own a restaurant and have ordered too much French wine, an efficient approach for selling that specific wine may involve playing French music in the background.^4^ The mental process behind the effect is called “priming”.^1^^,^^2^^,^^5^ Priming has been studied in many fields including political science,^6^ cognitive science,^7^^,^^8^^,^^9^^,^^10^ economics,^2^^,^^11^^,^^12^ psychophysiology,^13^ developmental psychology,^14^^,^^15^^,^^16^ psycholinguistics,^17^^,^^18^ law,^19^ education,^20^^,^^21^ philosophy of the mind,^22^^,^^23^ and business.^24^ When the effect is prolonged, priming relies on long-term memory,^25^ whereas if the effect is short-lived (e.g., seconds), priming relies on short-term memory.^26^ Here, we focus on short-term priming.

**Figure 1:**
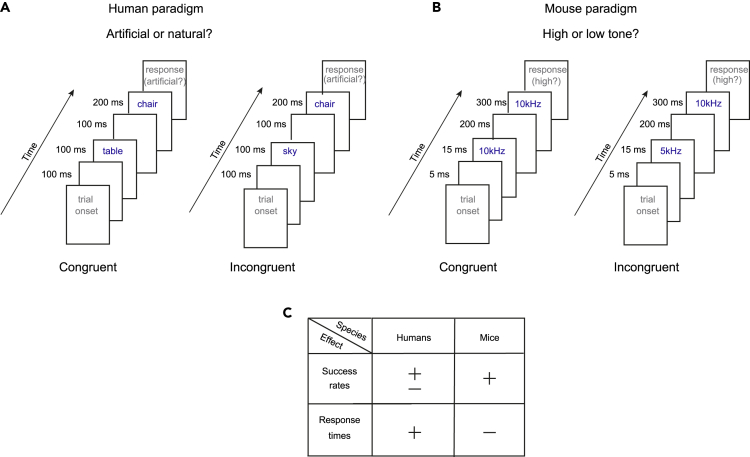
Paradigms for studying short-term priming in humans and in mice (A) Standard paradigm for testing short-term response priming in humans. **Left**, A typical timeline for a congruent prime trial (CPT). “Table” and “chair” are both pieces of furniture and belong to the same semantic content group. **Right**, A typical timeline for an incongruent prime trial (IPT). In the context of the experiment, the word “sky” (the prime stimulus) and the word “chair” (the target stimulus) do not belong to the same semantic content group. A “sky” target stimulus implies a “left” choice, and “chair” implies “right”. When the prime and target stimuli are congruent, human response times are typically shorter. (B) Proposed paradigm for testing short-term priming in mice. **Left**, A typical timeline for a CPT. The prime stimulus (short pure tone) and the target stimulus (long pure tone) belong to the same frequency group, since both tones have the same frequency, 10 kHz. **Right**, A typical timeline for an IPT. In the context of the experiment, 5 kHz and 10 kHz pure tones do not belong to the same content group. A target stimulus of “5 kHz” implies a “left” choice, and “10 kHz” implies a “right” choice. When the prime and target stimuli are congruent, mouse success rates are typically higher. (C) In humans, the typical priming effect is in response time. In contrast, we find that in mice the priming effect is in success rates, and not in response times.

Formally, priming is defined as a change in the mental processing of a stimulus as a result of prior encounter with a related stimulus.^27^^,^^28^ In the restaurant example, the type of music played would be the “prime” experience, and the type of wine selected would be the “target”. Multiple paradigms have been developed for investigating short-term priming.^28^^,^^29^^,^^30^^,^^31^ A distinction is made when the prime is processed consciously, i.e., the subject is aware of the prime stimulus, as opposed to unconscious processing.^28^^,^^32^^,^^33^^,^^34^ One specific paradigm is “response priming”,^35^^,^^36^ in which the prime and target stimuli are presented in quick succession and are coupled with identical or alternative responses. Response priming studies typically employ two alternative forced choice (2AFC) tasks for quantifying the influence of the prime on performance. Even when the subject is told that the prime is irrelevant for the performance of the task, the prime influences the outcome^28^ (Figure 1A). For example, the word “chair” (here, the target stimulus) is classified as “artificial” more rapidly when following the word “table” (the congruent prime stimulus; Figure 1A, left), as opposed to following the word “sky” (the incongruent prime stimulus; Figure 1A, right). The priming effect is quantified by a performance difference between congruent prime trials (CPTs) and incongruent prime trials (IPTs). Differences are typically evident in response times^27^^,^^32^^,^^37^ and may also be evident in success rates.^36^^,^^38^

Almost all research on priming has been conducted on human subjects. With human subjects, verbal explanations are possible, but not with animals. An extensive literature review revealed less than a dozen studies related to short-term priming in animals: birds,^39^^,^^40^^,^^41^^,^^42^^,^^43^ rats,^44^^,^^45^ lemurs,^46^ and monkeys.^47^^,^^48^ To date, there are no studies of priming involving cellular-level electrophysiology in humans or animals. Consequently, existing models of priming are phenomenological, including “global neuronal workspace”^49^ and "accumulator”^50^ models. There are no physiological mechanistic models for priming.^28^^,^^51^ For instance, priming may consist of a combination of behavioral repetition suppression and top-down influence of attentional amplification of task-congruent processing pathways.^52^ However, alternative mechanistic models are difficult to contest without access to cellular-network activity.

Here, we developed an auditory response priming paradigm for freely moving mice (Figure 1B). In two preliminary stages, we train the subjects to discriminate between the frequencies of long pure tones and to ignore short pure tones. We then couple short and long tones in the same trial. In contrast to studies of short-term priming in humans, we do not find any difference in response times. However, we find a consistent difference between success rates during CPTs and IPTs in all subjects. The results show that priming occurs in mice, suggesting that mice enjoy the same benefit as humans from the automatic activation of a first stimulus when performing a task on the second. Finally, we find that compared to same-session discrimination trials (DTs), success rates are reduced during IPTs more than during CPTs, suggesting that differential interferences underlie short-term priming in mice. The presented model system opens the door to studying the molecular, genetic, and cellular-network mechanisms at the basis of priming.

## Results

There are three features that set the priming phenomenon apart from other phenomena. The first feature is an association between the first (prime) stimulus and the second (target) stimulus, or the response. The second feature is the irrelevance of the prime stimulus to the response (the choice). And the third feature is the conflict between the prime-target (or the prime-response) association, and the irrelevance of the prime stimulus to the response.

**Figure 2:**
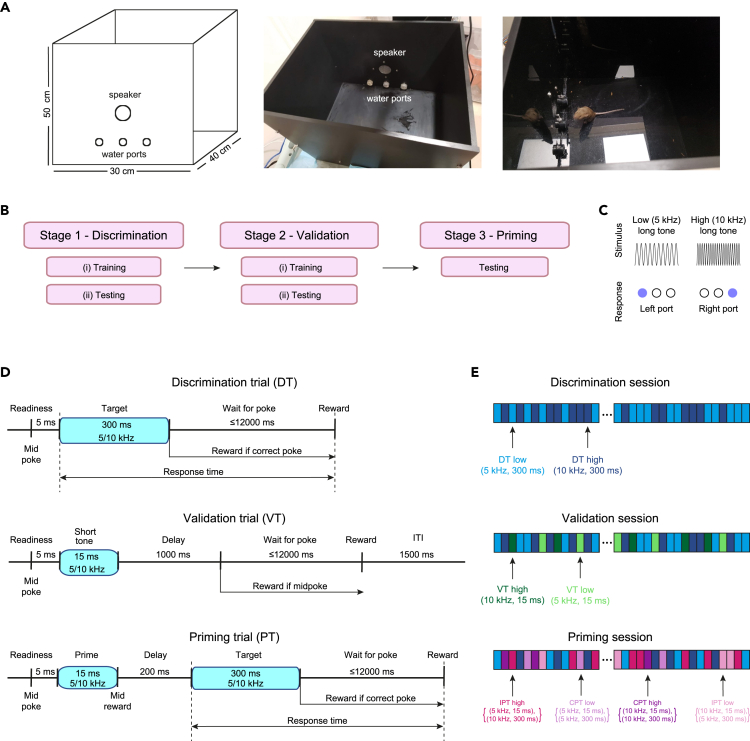
Apparatus and training procedure for testing short-term auditory priming in mice (A) The behavioral apparatus. One wall of the box contains all sensors and actuators, including a speaker and three illuminated water ports equipped with infrared LEDs for detecting nose pokes. (B) Mice are trained in two preliminary stages before being tested on the priming task. Stage 1 includes a simple discrimination task, in which the stimulus-response (tone-side) contingency is learned using long (300 ms) pure tones. Stage 2 includes a validation task, in which the mice learn that short (15 ms) pure tones are irrelevant for choosing a correct response. Stage 3 includes the priming task. (C) Stimulus-response contingency. In all tasks, a low (5 kHz) long pure tone requires a left nose poke, and a high (10 kHz) long pure tone requires a right nose poke. (D) Timelines for the three types of trials. **Top,** Discrimination trials (DTs). **Middle**, Validation trials (VTs). **Bottom**, Priming trials (PTs). ITI, inter-trial interval. (E) Example sequence of trials during discrimination, validation, and priming sessions. Every rectangle represents a single trial. See also Video S1 (an excerpt from a discrimination session), Video S2 (a validation session), and Video S3 (a priming session).

In the mouse priming paradigm, all behavioral training and testing sessions were carried out in the same apparatus (Figure 2A). Sessions were organized in three stages, each consisting of one or more distinct tasks (Figure 2B). The stimulus-response contingency was the same during all stages (Figure 2C). The first stage included discrimination sessions, composed of DTs (Figures 2D and 2E, top; Video S1). The DTs were designed to teach the animal an association between the frequency of the target stimulus and the response. Once the frequency-port association is learned, it is automatically applied to short tones as well, rendering the (short) prime stimuli relevant. Therefore, the second stage consisted of validation sessions, in which DTs were supplemented by validation trials (VTs; Figures 2D and 2E, middle; Video S2). In VTs, the animal had to learn to ignore short tones, while continuing to respond to long tone stimuli. Priming can be assessed only after the animal has learned the association between the long tones and the response, as well as the irrelevance of the short tones to the response. Thus, the third stage consisted of priming sessions, in which the DTs were supplemented by priming trials (PTs; Figures 2D and 2E, bottom; Video S3). To move from the first to the second stage, or from the second to the third stage, the animals had to fulfill a performance criterion. The third stage did not include training phases and only involved testing on the priming task.

**Figure 3:**
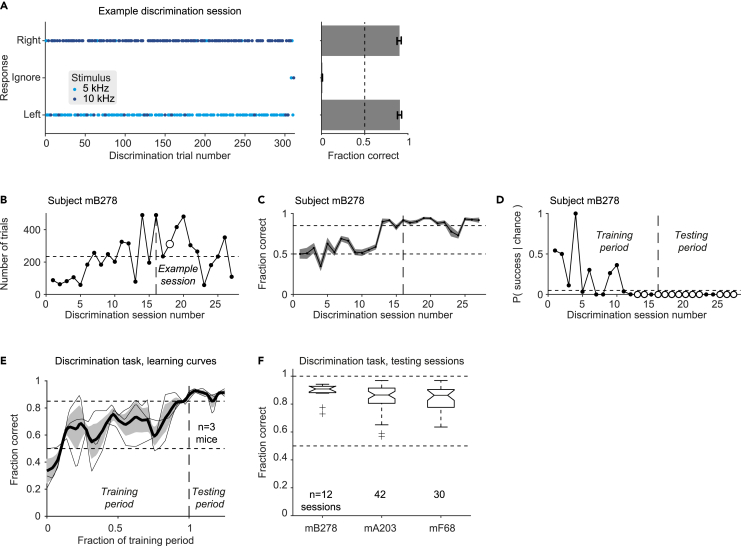
Mice learn to discriminate lower pure tones from higher pure tones (A) An example discrimination session performed by subject mB278. **Left**, Every dot represents a single DT. Dot color represents the target stimulus in the trial, i.e., the frequency of the 300 ms tone, which can be 5 kHz (light blue) or 10 kHz (dark blue). The vertical location represents the response (mouse choice) during the trial, which can be the left port (bottom), ignoring the tone (middle), or the right port (top). A session with perfect performance would yield a graph with all dark blue dots on top, all light blue dots at the bottom, and no dots in the middle. The example session consisted of 311 trials with a 90% success rate. The mouse ignored one low and one high tone, both toward the end of the session. **Right**, Fraction histograms for left correct trials (bottom), right correct trials (top), and ignored trials (middle). Error bars represent SEM. (B) The number of trials in every discrimination session carried out by one subject (mB278). Enlarged empty circle indicates the example session shown in A. (C) Success rate, defined as the fraction of correct DTs, for the same sessions as in B. Error bands represent SEM. (D) The p values of deviation from chance for subject mB278 sessions (Binomial test comparing to chance level, 0.5). Successful sessions (p < 0.05 and success rate above 0.85) appear as enlarged empty circles. In B–E, vertical dashed lines mark the first of four consecutive successful sessions, considered the point at which the mouse has learned the discrimination task. The same session is the first session of the testing period (F). (E) Learning curves for the three mice trained on the discrimination task. Thin lines show performance (fraction correct) of the individual mice (linear time warping) during the training period and for the beginning of the testing period. Thick line and patch show mean and SEM over mice. All mice start around chance level and perform consistently above 85% after training. (F) Post-learning performance of subjects mB278, mA203, and mF68 on the discrimination task. Every boxplot shows median and interquartile range (IQR) for one subject. Whiskers extend for 1.5 times the IQR in every direction, and a plus sign indicates an outlier. All mice learned the task, with an overall success rate of 88% [79% 91%] (median [IQR], n = 84 sessions).

Video S1. Subject mF68 performing the discrimination task (stage 1), related to Figures 2 and 3 In every trial, the pure tone stimulus is denoted as “Low DT” (5 kHz, 300 ms) or “High DT” (10 kHz, 300 ms). The response is denoted as “Correct”, “Incorrect”, or “Ignored”. For a 5 kHz / 10 kHz long tone, the correct response is poking the left / right port, respectively.

**Figure 4:**
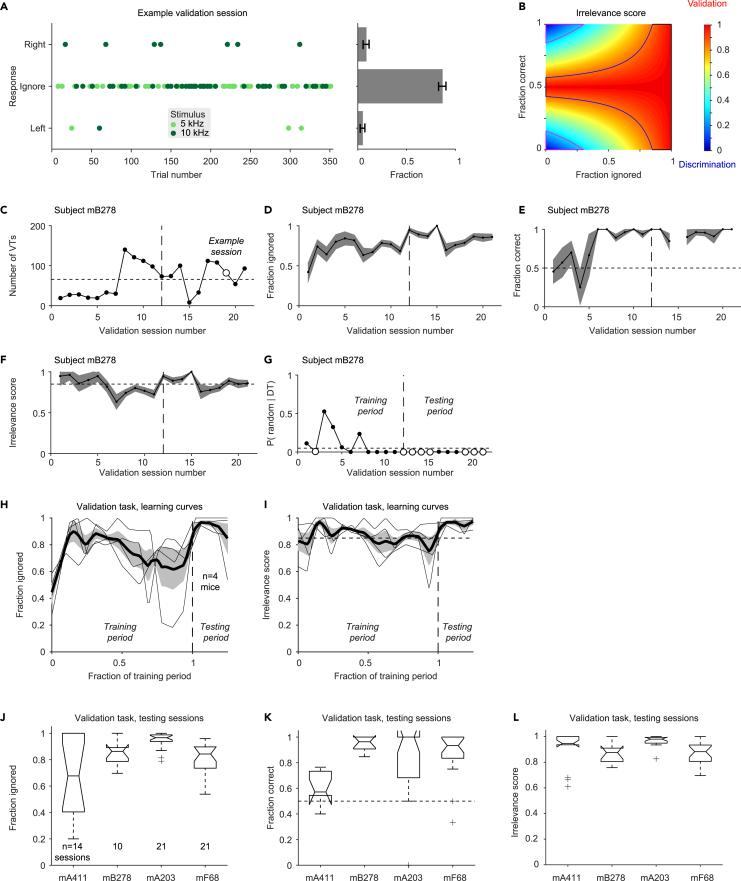
Mice learn to ignore short tones while discriminating long tones (A) All VTs in an example validation session of subject mB278. **Left**, Every dot represents a single VT. Dot color represents the frequency of the short pure tone (15 ms), which can be 5 kHz (light green) or 10 kHz (dark green). The vertical location of the dot represents the response (left port, ignorance, or right port). A validation session with perfect performance during VTs should yield a graph with all dots in the middle. **Right**, Fraction histograms for the number of the trials corresponding to every response. Error bars represent SEM. The subject ignored short tones during 71/82 (87%) of the VTs. Of the not-ignored VTs, the mouse made a “correct” choice in all three low short tones and in 7/8 of the high short tones. (B) Dependence of the “irrelevance score” on the fraction of ignored VTs and on success during not-ignored VTs. The score is defined as *irrelevance* = 1–2 *f*_*ni*_|0.5-*r*_*ni*_|, where *f*_*ni*_ is the fraction of not-ignored trials, and *r*_*ni*_ is the success rate during the not-ignored trials. When performance is very close to discrimination, scores fall in blue-hued parts of the graph, indicating that the subject has not learned the validation task. When the desired performance on the validation task is achieved, scores fall in the red-hued parts. (C) The number of VTs in every validation session carried out by one subject (mB278). Enlarged empty circle indicates the example session shown in A. (D) The fraction of ignored VTs, for the same sessions as in C. Here and in EFHI, error bands represent SEM. (E) The fraction of “correct” trials out of the not-ignored VTs. (F) Irrelevance scores. (G) The p values of irrelevance score for every validation session of subject mB278 (Monte Carlo test). Sessions with successful validation (p < 0.05 and irrelevance score above 0.85) appear as enlarged empty circles. In C–I, vertical dashed lines mark the first of four consecutive successful sessions, considered the point at which the mouse has learned the validation task. (H) Learning curves for the four mice trained on the validation task showing the fraction ignored. All conventions are the same as in Figure 3E. At the beginning of the training period, the mice ignore only some of the short tone stimuli. (I) Learning curves showing the irrelevance score. After training, the irrelevance scores are consistently above 0.85. (J) Fraction of ignored VTs, pooled over all testing sessions for subjects mA411, mB278, mA203, and mF68. Here and in **KL**, boxplot conventions are the same as in Figure 3F. (K) “Success rates” for the not-ignored VTs, pooled over all testing sessions for every mouse. (L) Post-learning irrelevance scores, pooled over all testing sessions for every mouse. All mice learned the validation task, with an overall irrelevance score of 0.94 [0.86 0.99] (median [IQR], n = 66 sessions).

Video S2. Subject mA203 performing the validation task (stage 2), related to Figures 2 and 4 In every trial, the pure tone stimulus is denoted as “Low DT” (5 kHz, 300 ms), “High DT” (10 kHz, 300 ms), “Low VT” (5 kHz, 15 ms), or “High VT” (10 kHz, 15 ms). The response is denoted as “Correct”, “Incorrect”, or “Ignored”. For a 5 kHz / 10 kHz long tone (300 ms), the correct response is poking the left / right port, respectively. For short tones (15 ms), the correct response is poking the middle port (ignorance).

**Figure 5:**
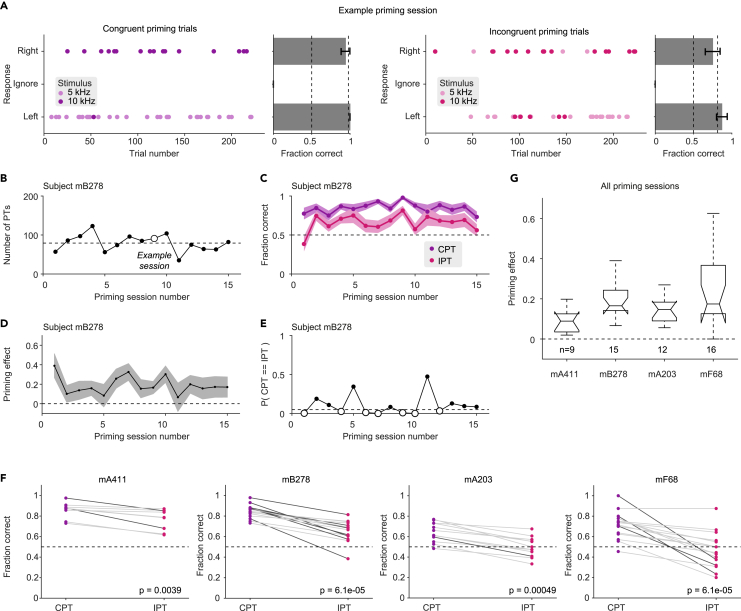
All mice show a priming effect in success rates (A) All PTs during an example priming session performed by subject mB278. Every dot indicates a single PT. Dot color represents the frequency of the target stimulus (300 ms tone), which can be 5 kHz (light-colored) or 10 kHz (dark-colored). The vertical location of the dot represents the response (choice), which can be the left port (bottom), ignorance (middle), or the right port (top). A priming session with perfect performance would yield a graph with all dark-colored (violet or pink) dots on top and all light-colored (violet or pink) dots at the bottom. **Left**, All congruent PTs (CPTs) during the session. **Right**, All incongruent PTs (IPTs). In all bar graphs, error bars represent SEM. Success rate is higher during CPTs (98%) compared with IPTs (81%; p = 0.011, permutation test). (B) The number of PTs during every priming session of subject mB278. (C) Success rates during the CPTs (violet) and during the IPTs (pink) of every session, for the same sessions as in B. Here and in D, error bands represent SEM. (D) The “priming effect” during every session of subject mB278, defined as the difference between the same-session CPT and IPT success rates. (E) The p values of the priming effect for every priming session (permutation test, comparing to an effect size of zero). Sessions with a significant (p < 0.05) priming effect appear as enlarged empty circles. (F) Success rates for all priming sessions performed by subjects mA411, mB278, mA203, and mF68. Every violet dot represents the success rate during the CPTs during one session and is connected by a line with a pink dot, representing the success rate during the same-session IPTs. p values, two-tailed Wilcoxon’s signed-rank paired test. (G) Priming effects for the four subjects. Boxplot conventions are the same as in Figure 3F. The overall priming effect is 15% [9.1% 20%] (median [IQR], n = 52 sessions).

Video S3. Subject mB278 performing the priming task (stage 3), related to Figures 2 and 5 In every trial, the pure tone stimulus is denoted as “Low DT” (5 kHz, 300 ms), “High DT” (10 kHz, 300 ms), “Low CPT” {(5 kHz, 15 ms), (5 kHz, 300 ms)}, “High CPT” {(10 kHz, 15 ms), (10 kHz, 300 ms)}, “Low IPT” {(10 kHz, 15 ms), (5 kHz, 300 ms)}, or “High IPT” {(5 kHz, 15 ms), (10 kHz, 300 ms)}. The response is denoted as “Correct”, “Incorrect”, or “Ignored”. For a 5 kHz / 10 kHz long tone, the correct response is poking the left / right port, respectively.

### Mice learn to discriminate lower pure tones from higher pure tones

The first stage of the learning process contained only discrimination sessions.^53^ In each DT, one of two pure tones was presented for 300 ms: a low target tone (5 kHz) or a high target tone (10 kHz). Both tones are in the audible range for mice^54^ and for humans. During the training period, the mouse had to associate the low-frequency tone with the left port, and the high-frequency tone with the right port. An example post-learning discrimination session can be found in Figure 3A. In the example session there were 154 trials with 5 kHz tones and 157 trials with 10 kHz tones. The mouse ignored two trials toward the end of the session and was incorrect in 29 other trials, yielding an overall success rate of 280/311 (90%).

Mice started the first discrimination sessions with success rates around 0.5. During training, the number of trials per session (Figure 3B) and the success rates (Figure 3C) gradually increased. All mice tested on the paradigm learned the discrimination task, achieving the criterion of at least four consecutive sessions with above-chance performance (p < 0.05, binomial test) and success rate above 85%. For the example subject, the transition from learning to post-learning is marked by a dashed vertical line in Figure 3D. The number of training sessions before learning was 15, 13, and 24, for subjects mB278, mA203, and mF68, respectively. After the learning period ended, additional discrimination sessions were conducted to measure and consolidate performance. Similar learning curves were observed in all three subjects (Figure 3E). During the post-learning sessions, all subjects exhibited consistently high performance (Figure 3F), with a median [interquartile-interval, IQR] post-learning success rate of 88% [79% 91%] (n = 84 sessions). The number of trials was self-paced by the mice, with a median [IQR] of 239 [171 322] trials per post-learning discrimination session. Subject-specific success rates were 91% [88% 92%] (n = 12 sessions, mB278); 87% [80% 91%] (n = 42, mA203); and 86% [76% 90%] (n = 30, mF68). Thus, all the mice achieved stable and high performance of discriminating between lower (5 kHz) and higher (10 kHz) pure tones.

### Mice learn to ignore short tones while discriminating long tones

After learning the discrimination task and exhibiting consistently high performance, the mice were trained to ignore short (15 ms) pure tones. A crucial element of every testable priming paradigm is that the subject will understand that the task should be performed on the target and not on the prime. With human subjects, verbal explanations are useful, but not with mice. To establish that short tones do not predict a correct choice of side port (right or left) and are therefore irrelevant to the response, VTs were used. In each VT, one of two pure tones was presented for 15 ms: a low short pure tone (5 kHz), or a high short pure tone (10 kHz). To suppress the tendency of mice pretrained on the stimulus-response contingency during DTs to choose a side port, a middle port reward was given if the mouse poked the middle port at least 1,000 ms after the short tone ended (Figure 2D, middle). During training, the mouse had to learn that there is no association between short tones and a choice of a side port. In practice, the subject had to respond to short tones at chance level, or to ignore the short tones and choose a side port only after long tones. In addition to VTs, every validation session contained DTs, identical to the DTs employed during discrimination sessions.

All the VTs of an example post-learning validation session are shown in Figure 4A. The example session included a total of 353 trials, of which 82 were VTs and the rest were DTs. Of the VTs, 38 trials included low short tones and 44 trials included high short tones. The mouse correctly ignored 35/38 (92%) of the low tones and 36/44 (82%) of the high tones. Thus, 71/82 (87%) of the VTs were ignored. During not-ignored trials, the “success rate” was 10/11 (91%). To take into account the fraction of the ignored trials as well as the responses during not-ignored trials, we defined an “irrelevance score” (Figure 4B). The irrelevance score is close to 1 when the short tones are ignored (Figure 4B, red) and close to 0 when the mouse fails to ignore the short tones (Figure 4B, blue). For the example validation session, the irrelevance score was 0.89.

The example subject (mB278) started training on the validation task after having learned the discrimination task to criterion. Thus, during the initial validation sessions the subject did not ignore the short tone stimuli (Figure 4D). However, during training, the fraction of ignored VTs gradually increased and stabilized (Figure 4D). The subject performed correctly during not-ignored VTs (Figure 4E), and the irrelevance score exhibited increases and decreases during training on the validation task (Figure 4F). Learning was defined as crossing a criterion of four consecutive sessions with above-chance irrelevance score (p < 0.05, Monte Carlo test) maintained above 0.85 (Figure 4G). For the example subject, the transition from learning to post-learning is marked by a vertical dashed line (Figures 4C–4G).

Similar learning curves were observed in all four subjects (Figures 4H and 4I). There were occasional high-performance sessions during training, and the learning process was not perfectly monotonic. The entire training period was required for performance to stabilize, possibly due to the conflict between the prime-target association and the irrelevance of the short tones. During training, subjects mA411, mB278, mA203, and mF68 required 13, 11, 8, and 16 validation sessions. After the training period ended, additional validation sessions were conducted to consolidate and measure performance on the validation task (Figures 4J–4L). The median [IQR] number of trials was 261 [173 366] per session, of which 52 [30 79] were VTs (n = 66 sessions in four mice). During the post-learning period, subjects mB278, mA203, and mF68 consistently ignored the short tones (Figure 4J). Subject mA411, which was trained on the discrimination and validation tasks in parallel, exhibited distinct behavior, performing closer to chance level during not-ignored VTs (Figure 4K). Regardless, all mice rendered the short tones irrelevant: subject-specific irrelevance scores were 0.95 [0.68 1.00] (n = 14 sessions, mA411); 0.88 [0.78 0.91] (n = 10, mB278); 0.98 [0.95 0.99] (n = 21, mA203); and 0.88 [0.77 0.93] (n = 21, mF68; Figure 4L). Overall, the post-learning irrelevance scores were 0.94 [0.86 0.99] (n = 66). Thus, all mice learned to ignore the short tone stimuli.

### All mice show a priming effect in success rates

The third stage contained priming sessions. Every priming session consisted of DTs, identical to the DTs employed during discrimination sessions, and PTs. Every PT included a short pure tone (the prime stimulus) followed by a long tone (the target stimulus; Figures 2D and 2E, bottom). PTs in which the short and long tones are of the same frequency are called congruent PTs (CPTs), whereas PTs in which the frequencies of the short and long tones differ are called incongruent PTs (IPTs). During PTs, the mouse was rewarded in the same manner as during DTs, namely for poking the right port following a low long tone, and for poking the left port following a high long tone (Figure 2C).

All PTs of an example priming session are shown in Figure 5A. The session included 234 trials, of which 91 were PTs and the rest were DTs. Of the PTs, 48 were CPTs (Figure 5A, left) and 43 were IPTs (Figure 5A, right). The mouse made a correct choice during 47/48 (98%) of the CPTs and during 35/43 (81%) of the IPTs. We quantified the “priming effect” in success rates by the difference between CPT and IPT success rates, which was 17% in the example session (p = 0.011, permutation test). Thus, in the specific case there was a consistent effect of the short tones on choice to the long tones: a short-term priming effect.

In contrast to the discrimination and validation tasks, we did not train the mice on any specific criterion during PTs. However, success rates were higher during CPTs compared with IPTs during all sessions of every mouse, with only one exceptional session (see in the following). For instance, subject mB278 exhibited a median [IQR] priming effect of 16% [10% 26%], with a range of [7%, 39%] (Figures 5C and 5D). The priming effect was significant only in some of the sessions (7/15 sessions with p < 0.05, permutation test; Figure 5E; dark gray lines in Figure 5F, second panel from left). However, a positive priming effect was observed in all sessions of the same subject (15/15; p < 0.001, Wilcoxon’s paired signed-rank test; Figure 5F, second panel from left). Similar results were observed in the other mice: success rates were consistently higher during CPTs compared with IPTs in all subjects (p < 0.005 in all cases; Figure 5F).

We conducted a total of 52 priming sessions in four mice, with a median [IQR] of 148 [91 199] trials per session, of which 71 [34 91] were PTs. There was a significant priming effect in 14/52 (27%) of the sessions (dark gray lines in Figure 5F). The effect was positive in 51/52 (98%) of the sessions. In one session, success rates were 88% during both CPTs and IPTs (Figure 5F, right panel). Subject-specific priming effects were reproducible over subjects (Table 1; Figure 5G). Thus, all mice exhibited a consistent priming effect, with a global priming effect of 15.1% [9.1% 20%] (median [IQR] over n = 52 sessions).

**Table 1:** Priming effect in every animal subject

Animal ID	Strain^a^	Sex	Age^b^ [weeks]	Weight^b^ [g]	Priming effect^c^
mA411	HYB	Female	8	19.4	8.9% [3.3 12.5]% (n = 9)
mB278	HYB	Male	12	30.5	16.5% [10.1 25.7]% (n = 15)
mA203	HYB	Male	32	32.8	14.7% [8.9 17.9]% (n = 12)
mF68	C57	Male	27	29	17.4% [12.5 35.6]% (n = 16)

a HYB, hybrid mice, the F1 generation of an FVB/NJ female and a C57-derived male.

b At the beginning of the training period, excluding the weight of the implant (mA203 and mF68).

c Median [IQR] (number of priming sessions).

### None of the mice show a priming effect in response times

**Figure 6:**
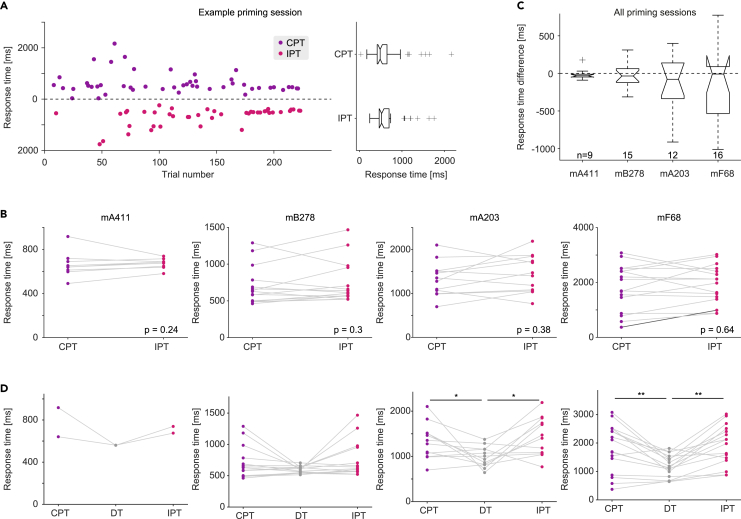
None of the mice show a priming effect in response times (A) Response times during all PTs carried out in an example priming session performed by subject mB278 (same session as described in Figure 5A). In a given PT, response time is defined as the duration from stimulus onset (target tone) to the response (side-port choice; Figure 2D, bottom). **Left**, The response times in each trial of the session. The bottom half of the graph corresponds to the response times in IPTs (pink) and the top half of the graph corresponds to the response times in CPTs (violet). **Right**, Response times during the example session are not consistently different between n = 48 CPTs and n = 43 IPTs (p = 0.15, Mann-Whitney U-test). Here and in C, boxplot conventions are the same as Figure 3F. (B) Median response times for all CPTs and IPTs during every priming session of every subject. p values, two-tailed Wilcoxon’s signed-rank paired test. Sample sizes and all other conventions are the same as in Figure 5F. (C) The differences of response times across all priming sessions for the four subjects. (D) Response times for all CPTs, DTs, and IPTs during every priming session of every subject. ∗/∗∗: p < 0.05/p < 0.01, two-tailed Wilcoxon’s signed-rank paired test. All other conventions are the same as in Figure 5F.

In human subjects, priming effects are typically observed in response times^28^^,^^55^^,^^56^ or in both response times and success rates.^36^^,^^38^^,^^50^ To determine whether mice exhibit a priming effect in response times in addition to success rates, we defined response time as the duration from target tone onset to side port selection (Figure 2D). For the same example session of subject mB278 described in Figure 5A, the median [IQR] response times were 487 [414 623] ms during n = 48 CPTs and 522 [460 697] ms during n = 43 IPTs (Figure 6A). During the example session, response times during CPTs and IPTs did not exhibit distinct medians (p = 0.15, U-test) or distinct variances (p = 0.49, permutation test). Response times during same-session DTs were 540 [481 594] ms (n = 143), not consistently different from (p = 0.19) or with distinct variance (p = 0.51) compared to the PT response times.

At the session level, response times were not consistently longer or shorter during CPTs compared with IPTs during any session of subject mB278 (p > 0.05 in all cases; n = 15 sessions; light gray lines in Figure 6B, second panel from left). At the subject level, there was no consistent difference between CPTs and IPTs in neither median response times (p = 0.3, Wilcoxon’s paired signed-rank test) nor the variance of response times (p = 0.79, permutation test; Figure 6B, second panel from left). Similar results were observed for the other three mice (session level: light gray lines in Figure 6B; subject level: p > 0.05 in all cases; Figure 6B). Of 52 priming sessions, we observed a consistent difference in response times between CPTs and IPTs in only one session (dark gray line in Figure 6B, right panel). The global difference in response times was −35 [-285 76] ms (median [IQR] over n = 52 priming sessions; Figure 6C). In sum, none of the mice exhibited a priming effect in response times.

The lack of a priming effect in response time may due to limited sensitivity of the priming task to response time. To test the possibility, we compared response times between same-session PTs and DTs for all subjects (Figure 6D). Over all priming sessions for which discrimination task data were available, the response times for all PTs (both CPTs and IPTs) were 1,146 [667 2051] ms (median [IQR] over 45 sessions). During same-session DTs, response times were 815 [645 1101] ms, yielding a median [IQR] intra-session difference of 232 [-20 694] ms (p < 0.001; Wilcoxon’s signed-rank test). Furthermore, PT response times were more variable compared with same-session DTs (p < 0.001; permutation test). Thus, response times differ between discrimination and PTs, but not between congruent and incongruent PTs.

### The prime stimuli exert an effect on success rate via differential interference

**Figure 7:**
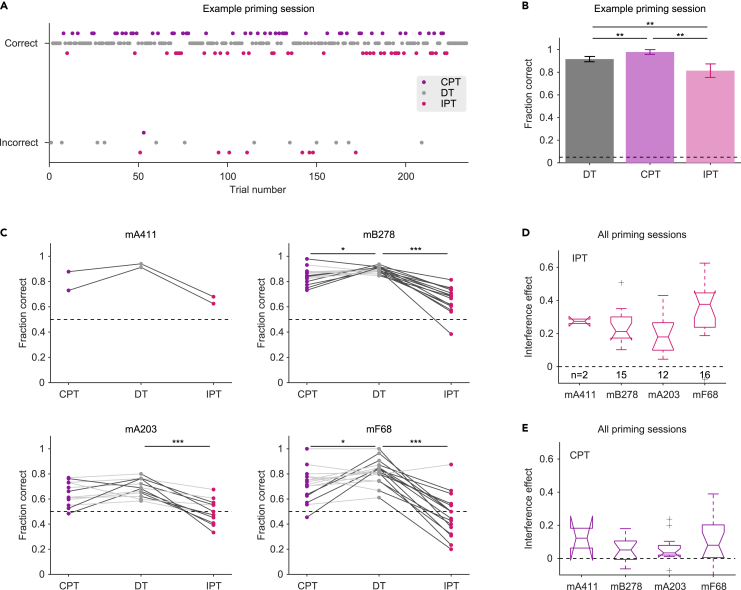
The prime stimuli exert an effect on success rate via differential interference (A) All trials during an example priming session of mB278 (same session as described in Figures 5A and 6A). CPTs, DTs, and IPTs are presented in a pseudorandom order. Trials with correct choices are shown on top, and trials with incorrect choices at the bottom. Stimulus frequency is not indicated. (B) Success rates during the three trial types for the session shown in A. Error bars represent SEM. ∗∗: p < 0.01, Monte Carlo test. (C) The success rates for all CPTs, DTs, and IPTs during every priming session of every subject. ∗/∗∗/∗∗∗: p < 0.05/p < 0.01/p < 0.001, two-tailed Wilcoxon’s signed-rank paired test. All other conventions are the same as in Figure 5F. (D) The interference effect during IPTs, defined as the session-specific difference between success rates during DTs and PTs. Here and in E, boxplot conventions are the same as in Figure 3F. (E) Interference during CPTs.

The specificity of the priming effect to success rates leaves open the question of whether congruent prime stimuli facilitate performance, incongruent primes interfere with performance, or both. Since success rates during DTs were not perfect (median [IQR]: 88% [79% 91%]; n = 84 discrimination sessions), there may be room for both improvement and deterioration. To contrast the possibilities, we compared performance in CPTs, IPTs, and DTs during the same session. In the example session of subject mB278, the animal made correct choices during 131/143 (92%) DTs, 47/48 (98%) CPTs, and 35/43 (81%) IPTs (Figure 7A). The success rates during CPTs were higher than during DTs (p = 0.005, Monte Carlo test; Figure 7B), and success rates during IPTs were lower than during DTs (p = 0.007). Thus, success rates during PTs may be either higher or lower than during same-session DTs.

To determine whether and in what direction performance is modified during CPTs and IPTs, we compared success rates at the subject level. For subject mB278, there was a consistent intra-session difference (p < 0.05, Monte Carlo test) between IPT success rates compared to DT success rates during all 15/15 (100%) priming sessions (p < 0.001, Wilcoxon’s paired test; dark gray lines at the right side of Figure 7C, top right panel). The median [IQR] interference was 21% [16% 31%] (n = 15 sessions). In contrast, the difference between same-session CPTs and DTs was consistent during 7/15 (47%) of the sessions (p = 0.03; dark gray lines at the left side of Figure 7C, top right panel) and the median [IQR] interference was 5.1% [-3.6% 11.6%]. Similar results were obtained for the other subjects (Figure 7C). Thus, although both facilitation and interference are observed during PTs, the dominant effect is interference.

Over all priming sessions for which data were available, the median [IQR] interference effect of the incongruent primes was 24% [17% 35%] (n = 45 sessions in four mice; Figure 7D). The incongruent primes modified success rates during 40/45 (89%) sessions. In all 40 sessions, the effect of IPTs was interference. The median effect of congruent primes was also interference, 5.4% [0.95% 12%] (Figure 7E). However, in two sessions of two different subjects there was consistent facilitation (mB278, 6.3%; mA203, 7.4%). Thus, incongruent primes exert stronger interference compared with congruent primes, suggesting differential interference as a cognitive mechanism for short-term priming in mice.

## Discussion

We developed a short-term response priming paradigm for mice. Four subjects were trained on the auditory task, and all mice exhibited a priming effect in success rate. However, none of the mice exhibited a priming effect in response times. The prime stimuli exerted their effect via differential interference, predominantly during incongruent PTs. The paradigm enables investigating the neurobiological basis of the short-term priming phenomenon with a set of tools applicable to mice.

### Task design and training procedures

A short-term response priming task must include three features: an association between the prime stimulus and the target stimulus (or the response), irrelevance of the prime stimulus to the response, and a conflict between the association and the irrelevance. In the present paradigm, the prime-choice association was spontaneous, building upon the natural similarity between short and long tones of the same frequency. Thus, after training the mice on the stimulus-response contingency (Figure 2C), the key feature that must be learned is the irrelevance of the short tones to the response. The tendency of mice trained on the stimulus-response contingency to choose a side port following a short tone had to be suppressed, and a middle port reward was utilized during VTs and maintained during PTs.

The addition of a middle port reward did not obscure the priming effect in success rates between CPTs and IPTs (Figure 5G) but likely contributed to the prolonged response times during PTs as opposed to DTs (Figure 6D), and thus alternative designs may be considered. One possibility could be to first train the animals on the irrelevance of all short tones using, e.g., a Go/No-Go paradigm. However, subsequent training on the stimulus-response contingency would extinct the irrelevance of short tones due to the natural similarity between same-frequency short and long tones, again requiring the usage of a middle port reward. More generally, because frequency and duration are two features of the played tone, learning one of them in stage 2 will extinct what was learned in stage 1. A second possibility is to punish the subject during PTs if a side port is selected before the target tone begins. Suppression of the natural tendency to select a side port would need to be established before initiating PTs, so punishment-based training on the validation task would also be required. Effective punishment can take the form of an electric shock, an air puff, or simply an annoying sound, but runs the risk of distracting the subjects, thereby reducing the regularity of behavior and the number of trials. A third possibility is to equate the conditions between the DTs and PTs by adding a middle port reward during DTs. However, the response time variability during PTs is not expected to be reduced.

Rendering the prime stimuli (short tones) irrelevant to the response involved training the subjects on the validation task, which may have caused the prime stimuli to acquire a negative association value and required weeks of training. One alternative design may involve categorically different prime stimuli, e.g., noise bursts of different pitches. This may help establishing a priming paradigm which cannot have repetition as one of its building blocks, since after the first exposure the short tones would not be novel any longer. A validation stage will then be required. A second alternative is to employ purely neutral stimuli (e.g., colors). However, neutral stimuli would not be naturally associated with the target stimuli and will require the additional learning of the association with the target stimuli in order to generate a priming effect. The irrelevance of the neutral stimuli will still have to be learned, again requiring training on the validation task, with either negative or positive reinforcement.

To summarize, in a short-term response priming paradigm, the prime stimuli must be initially associated with the target stimuli (or with the choice). The association can be learned or natural. However, with animals, the association requires a validation period to establish irrelevance of the prime stimuli to the choice before priming can be assessed.

### Comparison to human studies

In most human response priming paradigms, the priming effect is detected in response time.^28^^,^^31^^,^^57^^,^^58^ Success rates remain at the ceiling and are unmodified by the prime-target congruency. In other studies, the priming effect is found in both success rates and response times.^36^^,^^38^ Then, the typical effect of the prime stimuli on success rates is reduction during IPTs, without modifying the already-perfect performance during CPTs.^50^ Thus, response times are more sensitive than success rates in human response priming studies.

In the present mouse experiments, we found a priming effect only in success rates, providing two qualitative differences between human^36^^,^^38^^,^^50^ and mouse performance (Figure 1C). First, in mice, the priming effect is in success rates and not in response times, whereas in humans, the priming effect is both in success rates and response times. The fact that success rates exhibited a clear priming effect may suggest that response time is not a necessary metric. Second, in mice, the main effect of the prime stimuli is interference, whereas in humans the main effect is interference following incongruent prime stimuli and improvement (or no effect) following congruent primes.

The first difference, namely the lack of an observed priming effect in mouse response times between congruent and incongruent PTs may stem from the different task requirements or from the different setting and apparatus used for humans and mice. Physical requirements differ, since moving your entire body toward the correct port in every trial demands much more energy compared to pressing a button. For both discrimination and PTs, the port visited in the previous trial may be reflected in body orientation at the beginning of a new trial, increasing mouse response time variability compared to humans. Variability was even higher during mouse PTs compared with DTs, due to the increased cognitive load during the processing of two stimuli as opposed to one, the consumption of the middle port reward, or other sources. Alternatively, it is possible that even when performing the exact same task with the exact same settings, humans and mice would have different time constants and exhibit different temporal variability.

The second difference, namely that the main effect of the prime stimuli on mouse performance is interference may indicate that the mere presence of the additional prime stimulus distracts and hinders mouse behavior more than human behavior. Perceiving the prime as a distractor may stem from differences in some cognitive abilities between humans and mice. For instance, humans and mice may assign different attentional weights to different kinds of events and may have different attitudes to the concept of a behavioral experiment.

### Extension to non-auditory, multi-modal, and unconscious priming

The current paradigm involves a specific auditory intra-modal short-term response priming task. However, the apparatus employed for the task was designed to be suitable for various extensions. First, the structure of the apparatus enables the development of a visual priming task in mice. For instance, blue light may be associated with left port choice, and green light with right port choice. Second, the apparatus also allows the potential study of inter-modal priming,^29^^,^^58^^,^^59^ in which the prime stimulus would be auditory, and the target stimulus would be visual, or vice versa. As opposed to the natural association between short and long tones of the same frequency, the prime-target association will have to be learned for inter-modal priming because colors are not naturally associated with tones. Third, the apparatus allows extending the conscious priming paradigm to an unconscious priming paradigm,^60^^,^^61^^,^^62^ for instance, by using white noise to provide forward and/or backward masking of the prime stimuli.

Priming paradigms that include masks allow testing the influence of different types of unconscious processing on conscious performance.^63^^,^^64^^,^^65^ Indeed, unconscious priming paradigms provide a window for researching unconscious processing pathways that subjects cannot report. Because of the relative complexity of the paradigm, unconscious short-term memory is presently studied almost only in humans, limiting the questions that can be asked and mechanisms that can be studied. Thus, future extensions of the present mouse priming paradigm may allow studying unconscious processing in rodents.

A key issue during the study of unconscious processing is the necessity to verify that the stimulus was not perceived. With humans, the verification may involve a subjective report, but with animals, other methods are necessary. The mouse priming task can be extended to study unconscious processing as follows. Referring to Figures 2B and 2D, trials during discrimination sessions (stage 1) can be supplemented with two consecutive auditory white noise masks before the target (long) stimulus. During PTs, the same white noise masks can be split into two stimuli that flank the prime (short) stimulus. Because mice previously trained on the discrimination task typically choose a side port when initially exposed to short tones (Figures 4D and 4H), a lack of response to short tone can be interpreted as efficient masking, or unconscious processing. Then, the validation task (stage 2) can be replaced with a calibration task in which the short tones are preceded and followed by the same white noise masks, omitting the long tones. By modifying the duration of the short tones and quantifying success rates, the longest ignored tone duration can be determined for every subject and utilized as the duration of the prime stimuli in the priming task (stage 3). An unconscious priming paradigm may enable the study of neural correlates of consciousness in freely moving mice.

### Using the rodent paradigm to study the neurobiological basis of priming

The neurobiological basis of sensitization, habituation, adaptation, and explicit short-term memory has been studied extensively.^66^ However, priming uniquely requires (1) an association between the prime stimulus and the target stimulus (or the response), (2) irrelevance of the prime stimulus to the response, and (3) a conflict between the association and the irrelevance. Indeed, several types of memory, including implicit short-term memory, have not been studied with invasive techniques, and their neurobiological basis is yet unknown.

Freely moving mice have emerged as a robust model for studying various neuronal mechanisms using high-density silicon probe recordings combined with optogenetics.^67^^,^^68^^,^^69^ Here, we created a conscious short-term response priming paradigm for mice. We tested the paradigm and found a consistent priming effect of success rate in all subjects, and the key features of the human priming phenomenon were observed in the mice. The paradigm is suitable for freely moving mice but can be adapted to other animal species and to head-fixed subjects, which is less organic but may provide reduced variability and an increased number of trials. We propose that the paradigm and the future extensions thereof provide a platform for researching the neurobiological mechanisms underlying short-term priming, using tools including genetic targeting, molecular analyses, and cellular-network electrophysiology. As such, the present work forms a necessary first step for investigating the intracortical cellular-network level basis of short-term priming in particular, and of implicit short-term memory in general.

### Limitations of the study

Several limitations must be noted. First, we employed a fixed duration of the post-prime delay during PTs. In human priming studies, the stimulus onset asynchrony (SOA) has a considerable influence on the outcome,^50^ and introducing variability with mice may reveal a priming effect in response times. Notably, large SOA variability may confuse the mice and reduce success rates. Second, we used a middle port reward to render and to maintain the prime stimuli irrelevant to the response. While a priming effect in success rate was clearly observed in all subjects, licking and reward-related neuronal activity involve widespread neuronal activation^70^^,^^71^^,^^72^^,^^73^^,^^74^ and may challenge detecting neuronal correlates of priming. However, previous work showed that small neuronal signals riding on a large background can still be detected,^69^^,^^72^^,^^75^ and neuronal signals associated with multiple features may be demultiplexed.^72^^,^^73^^,^^75^^,^^76^

## STAR★Methods

### Key resources table

**Table:** 

REAGENT or RESOURCE	SOURCE	IDENTIFIER
**Experimental models: Organisms/strains**		

Mouse: FVB/NJ	The Jackson Laboratory	RRID:IMSR_JAX:001800
Mouse: C57BL/6J	The Jackson Laboratory	RRID:IMSR_JAX:000664
Mouse: PV-Cre	The Jackson Laboratory	RRID:IMSR_JAX:008069
Mouse: CaMKII-Cre	The Jackson Laboratory	RRID:IMSR_JAX:005359
Mouse: Ai32	The Jackson Laboratory	RRID:IMSR_JAX:012569

**Software and algorithms**		

MATLAB	Mathworks	http://www.mathworks.com

**Other**		

Digital signal processor	Tucker-Davis Technologies	RX8

### Resource availability

#### Lead contact

Further information and requests for resources should be directed to and will be fulfilled by the lead contact, Eran Stark (eranstark@sci.haifa.ac.il).

#### Materials availability

This study did not generate new unique reagents.

### Experimental model and study participant details

#### Animals

Four freely-moving adult mice of both sexes were used in this study (Table 1). The first three subjects were hybrid mice. Hybrid mice were used since compared to progenitors, hybrids exhibit reduced anxiety-like behavior and improved learning.^68^ The first subject (mA411) was a hybrid female mouse, generated by crossing an FVB/NJ female (JAX #001800, The Jackson Laboratory) and a C57BL/6J male (JAX #000664). The second and third subjects, mB278 and mA203, were single-transgenic hybrid males, generated by crossing an FVB/NJ female with a parvalbumin (PV)-Cre male (JAX #008069). mB278 and mA203 were injected with a viral vector expressing Jaws^77^ (rAAV8/hSyn-Flex-Jaws) in neocortex and hippocampus as previously described.^78^ The fourth subject, mF68, was a dual-transgenic male, generated by crossing a CaMKII-Cre female (JAX #005359) with an Ai32 male (JAX #012569). In subjects mA203 and mF68, electrophysiological recordings and optical manipulations were carried out during some sessions. Recordings were carried out using a silicon diode-probe mounted on a micro-drive as previously described.^78^ Results of electrophysiological recordings and optogenetic manipulations are not included in the present report. All behavioral results were observed at the subject level (Figures 3, 4, 5, 6, and 7), and no differences were observed between implanted (mA203 and mF68) and un-implanted (mA411, mB278) subjects. All animal handling procedures were in accordance with Directive 2010/63/EU of the European Parliament, complied with Israeli Animal Welfare Law (1994), and approved by the Tel Aviv University Institutional Animal Care and Use Committee (IACUC #01-16-051 and #01-21-012).

### Method details

#### Apparatus

All behavioral sessions were conducted with the subject inside an audio-visual behavioral apparatus (Figure 2A). The apparatus consists of a 30 x 40 x 50 cm (L x W x H) open-top black-colored Plexiglas box. The front wall of the box hosts all sensors and actuators, arranged as three transparent behavioral ports. A speaker (22TAF/G, SEAS Prestige) for audio stimuli is located above the middle port. Every port contains a white LED for visual signaling; an infra-red LED-phototransistor pair for detecting nose pokes; and a steel tube connected via a flexible (Tygon) tube to a solenoid valve (003-0141-900, Parker) for water delivery. The solenoids were calibrated periodically to enable micro-liter resolution control of the amount of water dispensed.

The apparatus is controlled by a behavioral controller (BC). The BC is a metal enclosure containing a microcontroller (Arduino Mega) connected to custom-made electronic circuitry driven by a grounded power supply. Relevant digital events (sensor detection, actuator action, flow control) are routed from the BC via a multi-wire cable to the electrophysiology data acquisition system (DAQ) and to a real-time digital signal processor (DSP; RX8, Tucker-David Technologies). The microcontroller is connected to a PC via USB, and a custom graphical user interface is used to program the controller and report events to the experimenter. The direct communication between the BC, the DAQ, and the DSP allows precise temporal alignment of auditory stimuli, behavior, electrophysiological activity, and optogenetic stimuli.

#### Water deprivation during training and testing

Throughout the behavioral training and testing period, the mice were kept under a water deprivation protocol during weekdays, and received free water during weekends. During weekdays, every mouse received at least 40 ml/kg of water (i.e., 1 ml/day for a 25 g mouse). Body weight was maintained above 85% at all times but because mice were allowed to perform trials until losing interest, water consumption varied between sessions. Animal performance and the number of trials performed during a given session depend on body weight, but other factors may induce variability (e.g., Figures 3B and 4C). Some are well controlled (e.g., experimenter identity, time in day) whereas others are not (e.g., experimenter mood, behavior of other mice in the colony during the night).

During every behavioral session, the mouse was placed in the apparatus. The LED in the middle port was used to indicate trial onset to the mouse, and all three ports were used to quantify performance and govern the tasks in real time. At the beginning of the training period of the discrimination task (Figure 2B), the reward volume was 10 μl for every successful trial, which was gradually decreased down to 6 μl. Subsequently, the water reward volume was the same during all tasks and at all ports.

#### The discrimination task

The first stage of the learning and testing process (Figure 2B) contained discrimination sessions. Every session contained two types of discrimination trials (DTs; Figures 2D and 2E, top; Video S1). The target stimuli were 300 ms pure tones with frequencies of 5 kHz (“low target tone”) or 10 kHz (“high target tone”). The average binaural level, estimated at the middle of the head of the mouse while poking the middle port, was 65-70 dB SPL (measured using 4939-A-011 ¼” free field microphone, 2670 preamplifier, and 1708 conditioning amplifier; Brüel & Kjaer). The level followed previous work^53^ and was not adjusted or modified between experiments or mice. Correct performance involved the mouse poking a side port according to the tone played: the left port for the low target tone, and the right port for the high target tone (Figure 2C). The stimulus-response contingency was the same for all mice. Before every trial, the LED of the middle port was on. To initiate a trial, the mouse had to poke the middle port to indicate readiness (Figure 2D, top). 5 ms later, the target tone was played, the middle port LED turned off, the two side port LEDs turned on, and the system waited for the mouse to make a choice. If the mouse poked a side port or if 12000 ms elapsed without a side poke, the two side port LEDs turned off, the middle port LED turned on, and the system waited for the mouse to initiate the next trial. A correct choice of a side port resulted in water reward in that port. Lack of choice or an incorrect choice ended the trial without a water reward.

#### The validation task

The second stage of the learning and testing process consisted of validation sessions (Figure 2B). With human subjects, the irrelevance of the prime stimulus for correct performance of the task can be explained verbally but with animals, verbal explanations are not possible. To “explain” to the mouse the irrelevance of the priming stimulus for choosing a side port, we established a second learning stage termed “validation”. The purpose of the validation stage is to ensure that the mouse will understand that the short (prime) stimuli are not informative and should be ignored, and will keep responding to the long (target) stimuli.

The validation sessions consisted of a 3:1 pseudorandom mixture of DTs and validation trials (VTs; Figures 2D and 2E, middle; Video S2). The DTs were identical to the DTs employed during discrimination sessions, in which tone durations were 300 ms. In contrast, the tones in every VT were 15 ms pure tones with frequencies of 5 kHz (“short low tone”) or 10 kHz (“short high tone”). During a validation session, the mouse should ignore the short tones during the VTs, and respond to the long tones during the DTs exactly as during discrimination sessions. Ignoring the short tones can be done either by continued poking of the middle port until the next trial is initiated, or by leaving the middle port without selecting a side port. An alternative desirable response during VTs is random side port selection, i.e., a situation in which the not-ignored “success rate” is close enough to 0.5.

To initiate a VT the mouse had to poke the illuminated middle port, exactly as during a DT. Throughout the VT, the middle port LED was kept on and the two side port LEDs were kept off. 5 ms after the VT was initiated, the short tone was played for 15 ms (Figure 2D, middle) and the system waited for at least 1000 ms. If the mouse poked the middle port after the 1000 ms period has ended but before an additional 12000 ms have elapsed, a reward was given at the middle port. After a reward and a 1500 ms inter-trial interval, the mouse could initiate the next trial. Alternatively, if the mouse poked a side port at any point during the 13000 ms interval, the trial ended without a water reward. To prevent extinction of the discrimination task, a VT could only be followed by a DT (Figure 2E, middle).

Before training subjects on the validation task, we established the discrimination task by training several other mice only on DTs; these mice were not tested on the priming task and are not included in the present report. The first subject trained on the discrimination and validation tasks before being tested on priming was subject mA411. However, in contrast to the three subsequent mice (mB278, mA203, and mF68), mA411 was trained on the discrimination and validation tasks in parallel, during the same 27 sessions and is therefore excluded from the report in Figure 3. Because we suspected that parallel training may be inferior to sequential training, we shifted to sequential training in the following three mice. However, whether parallel training is inferior or superior to sequential training remains to be established.

#### The priming task

The third stage of the process contained priming sessions. Every priming session contained a 3:2 pseudorandom mixture of DTs and priming trials (PTs; Figures 2D and 2E, bottom; Video S3). The DTs were identical to the DTs employed during discrimination sessions. Every PT consisted of a pair of (prime, target) stimuli: the prime stimulus (short 15 ms tone, 5 kHz or 10 kHz) was followed by a target stimulus (300 ms tone, 5 kHz or 10 kHz; Figure 2D, bottom). Thus, PTs could be either congruent (CPT; Figure 1B, left) or incongruent (IPT; Figure 1B, right). During a CPT, the stimulus pair had identical frequencies, {(5 kHz, 15 ms), (5 kHz, 300 ms)} or {(10 kHz, 15 ms), (10 kHz, 300 ms)} for left or right CPTs, respectively. During an IPT, the stimulus pair had different frequencies, {(10 kHz, 15 ms), (5 kHz, 300 ms)} or {(5 kHz, 15 ms), (10 kHz, 300 ms)} for left or right IPTs, respectively.

A PT began when the mouse poked the illuminated middle port. 5 ms later, the prime stimulus (a short 15 ms pure tone) was played. To prevent extinction of the validation task, a reward was given in the middle port at the same time. After a 200 ms delay, the target stimulus (a 300 ms pure tone) was played, the middle LED turned off, the two side port LEDs turned on, and the system waited up to 12000 ms for the mouse to make a choice. Thus, stimulus onset asynchrony (SOA) was fixed at 215 ms. If the mouse poked the correct side port, a water reward was given in the same port. When the mouse poked a side port, the side LEDs immediately turned off, the middle LED turned on, and the system waited for the mouse to initiate a new trial by a poking the middle port. The new trial could be a DT or a PT (Figure 2E, bottom). If the mouse poked a side port before the target stimulus has been played, no reward was given, the LEDs reset, and the mouse could initiate a new trial.

#### Real-time countering of automated strategies

During learning of a new 2AFC task, mice tend to develop automated choice strategies which allow obtaining a reward in about 50% of the trials without actually learning the task.^53^^,^^79^ Possible automated strategies include alternation (choosing the right and left ports alternately), perseveration (e.g., choosing the right port in every trial), win-stay/loose-shift, and win-shift/loose-stay. In preliminary experiments with other subjects, we mainly observed alternation behavior. To prevent subjects from developing automated strategies during the discrimination task, we used online prediction of the automated strategy that the mouse is most likely to be taking. The algorithm (https://github.com/cjstoneking/antibias) estimated the instantaneous strategy and provided trials that minimize the effectiveness of the strategy. The procedure ensured that a mouse will not be able to quench its thirst without correctly performing the task.

### Quantification and statistical analysis

#### Performance during the discrimination task

We defined successful performance in a given discrimination session if (1) performance was above chance (Binomial test comparing to chance level of 0.5), and (2) the fraction of correct responses out of all (ignored and not-ignored) trials was above 0.85. We state that the mouse has finished the training phase and has learned the discrimination task (Figure 2B) when at least four successful sessions were performed consecutively. Measurements of post-learning performance (testing sessions) were taken from the first of the four sessions and onwards.

#### Performance during the validation task

To quantify performance in the validation task, we introduced an irrelevance score (Figure 4B). The score quantifies the extent to which short tones are effectively ignored by the subject, and can be interpreted as how well the subject “understands” that short tones are irrelevant to the discrimination task. Typically, a mouse well-trained on the discrimination task that is exposed to the validation task for the first time, responds to the short tones as to long tones and pokes a side port immediately. During training on the validation task, the mouse is required to learn to ignore the short tones. The irrelevance score is defined as:

$$\text{irrelevance}=1-2\;f_{\text{ni}}\left|0.5-r_{\text{ni}}\right|$$

where *f*_*ni*_ is the fraction of trials that were not ignored and *r*_*ni*_ is the “success rate” during not-ignored trials. The irrelevance score is 1 if all short tones are ignored (*f*_*ni*_=0) or if “success rate” during all not-ignored trials is 0.5 (*r*_*ni*_=0.5; Figure 4B, red). The irrelevance score is 0 if the mouse did not ignore any of the short tones (*f*_*ni*_=1) and has a not-ignored “success rate” of either *r*_*ni*_=0 or *r*_*ni*_=1 (Figure 4B, blue).

In validation sessions, we defined chance level performance as a stimulus-dependent choice pattern during the VTs that was identical to the choice pattern during the DTs of that specific session. The rationale is that the default behavior of a mouse that has mastered the discrimination task and is exposed for the first time to a validation task is to treat short tones as target tones. We defined validation performance in a single session as successful if the irrelevance score was (1) above “chance” (Monte Carlo resampling test, comparing to chance level score based on the same-session DTs), and (2) above 0.85. The Monte Carlo resampling involved 10000 iterations, in which choices during randomized VTs were drawn from the empirical distribution of the stimulus-dependent choices during same-session DTs. We state that the mouse has finished the training phase and learned the validation task (Figure 2B) when at least four successful validation sessions have been performed consecutively. Measurements of post-learning performance (testing sessions) were taken from the first of the four sessions and onwards.

#### Performance during the priming task

The discrimination and validation tasks were designed to train animals on a specific stimulus-response association, and training continued until performance stabilized and specific criteria were achieved (Figure 2B). In contrast, the priming task only involved testing the effect of short tones on the discrimination of long tones, and no performance criteria were set. A second difference is that the effect of the prime stimuli on discrimination during the priming task cannot be characterized at the single-trial level (correct/incorrect), but rather at the session level. We defined a “priming effect” in success rates as the difference between success rates during CPTs and success rates during same-session IPTs. To determine statistical significance, we used a permutation test, shuffling the labels of the CPTs and IPTs and comparing the observed priming effect (difference of success rates) with the null distribution of the priming effects obtained by 10000 iterations.

#### Statistical analyses

In all statistical tests, a significance threshold of α=0.05 was used. All descriptive statistics (n, median, IQR, mean, SEM) can be found in the Results, figures, and figure legends. For DTs, we used a one-tailed Binomial test comparing to chance level, 0.5. For VTs, we used Monte Carlo randomization (10000 iterations), drawing choices from the empirical distribution of same-session DTs. For PTs, we used a permutation test (10000 iterations), shuffling the labels between same-session CPTs and IPTs. Differences between medians of two paired groups were tested using Wilcoxon’s signed-rank test (two tailed). Differences between medians of two unpaired groups were tested using Mann-Whitney’s U-test (two tailed). Differences between the variances of two groups were tested using a permutation test (two tailed). All statistical analyses were conducted in MATLAB (MathWorks). For all figures, ∗, p<0.05; ∗∗, p<0.01; ∗∗∗, p<0.001.

## Data Availability

• All data reported in this paper will be shared by the lead contact upon request. • This paper does not report original code. • Any additional information required to reanalyze the data reported in this paper is available from the lead contact upon request.
